# Local soft tissue dissemination of large joint septic arthritis: A report of two cases

**DOI:** 10.1016/j.ijscr.2025.112098

**Published:** 2025-10-24

**Authors:** Ojochonu David Anthony, Jevan Cevik, Nelson Low

**Affiliations:** a Department of Plastic and Reconstructive Surgery, Monash Health, Dandenong Hospital, Melbourne, Australia

**Keywords:** Septic arthritis, Necrotising fasciitis, Soft tissue infections, Musculoskeletal infections, Surgical debridement, Case report

## Abstract

**Introduction:**

Septic arthritis (SA) is a surgical emergency that can result in joint destruction and systemic sepsis. Local soft tissue dissemination of native joint SA leading to necrotising fasciitis (NF) is a rare but devastating complication associated with poorer outcomes.

**Case presentation:**

We present two adult patients with poorly controlled diabetes who developed SA complicated by NF—one affecting the knee and posterior leg, the other the shoulder and anterior arm. Both required multiple joint washouts, serial wound debridements, intensive care unit (ICU) admission, and prolonged intravenous antibiotics. One patient sustained extensive soft tissue loss necessitating coverage with a biodegradable temporising matrix (BTM) followed by split-thickness skin grafting.

**Clinical discussion:**

These cases highlight the clinical course of this rare but severe complication and emphasise the importance of early diagnosis, initiation of broad-spectrum intravenous antibiotics, and timely surgical intervention. We also outline a practical diagnostic and management framework, including antimicrobial selection post-arthrocentesis, the role of pre-operative imaging, and the rationale for selecting arthroscopic versus open joint debridement.

**Conclusion:**

SA complicated by NF is a life- and limb-threatening condition that presents numerous diagnostic and therapeutic challenges. Early recognition and a multidisciplinary approach incorporating empirical antibiotics, targeted imaging, and prompt surgical management are essential to optimise outcomes.

## Highlights

• Septic arthritis can disseminate locally, causing necrotising fasciitis.
• Local spread of joint infection is rare but carries high morbidity.
• Diabetic and immunosuppressed patients are at greatest risk.
• Delayed diagnosis worsens outcomes in this elusive complication.
• A prompt multidisciplinary approach is required for optimal care.

## 1 Introduction

Septic arthritis (SA) is a surgical emergency that can lead to devastating outcomes, primarily through bacteraemia and systemic sepsis. However, local dissemination of the infection may also precipitate complications such as necrotising fasciitis (NF), myositis, tenosynovitis, and osteomyelitis. These often require extensive surgery, prolonged antibiotics, and are associated with increased mortality and disability [1,2]. Extensive soft tissue involvement often results in large defects that require reconstructive procedures, ranging from skin grafting to free flap surgery [3].

Local extra-synovial extension of SA resulting in NF in adults is rare, with few cases documented in the literature. In line with the SCARE criteria [4], we present two patients who presented to our community hospital with this condition, both requiring multiple operations and intensive medical management. Both cases illustrate the importance of early diagnosis, thorough debridement, and appropriate antimicrobial therapy. This article aims to highlight the clinical course of this major complication while suggesting a framework for optimal management.

## 2 Case 1

A 44-year-old male with poorly controlled type 2 diabetes mellitus presented to the emergency department with one week of right knee pain and swelling, accompanied by vomiting, fevers, and chills.

Examination revealed an irritable right knee; however, plain radiographs were unremarkable. Laboratory investigations showed markedly elevated inflammatory markers, acute kidney injury, and hyperglycaemia. Gram staining of a joint aspirate demonstrated Gram-positive cocci, signifying SA and prompting the initiation of intravenous piperacillin-tazobactam.

Urgent arthroscopic knee washout revealed frank pus, inflamed synovium, and minor degenerative changes. Postoperatively, he required intensive care unit admission for septic shock. Cultures from the aspirate and intraoperative samples confirmed Group A Streptococcus, and intravenous benzylpenicillin was commenced.

**Fig. 1:**
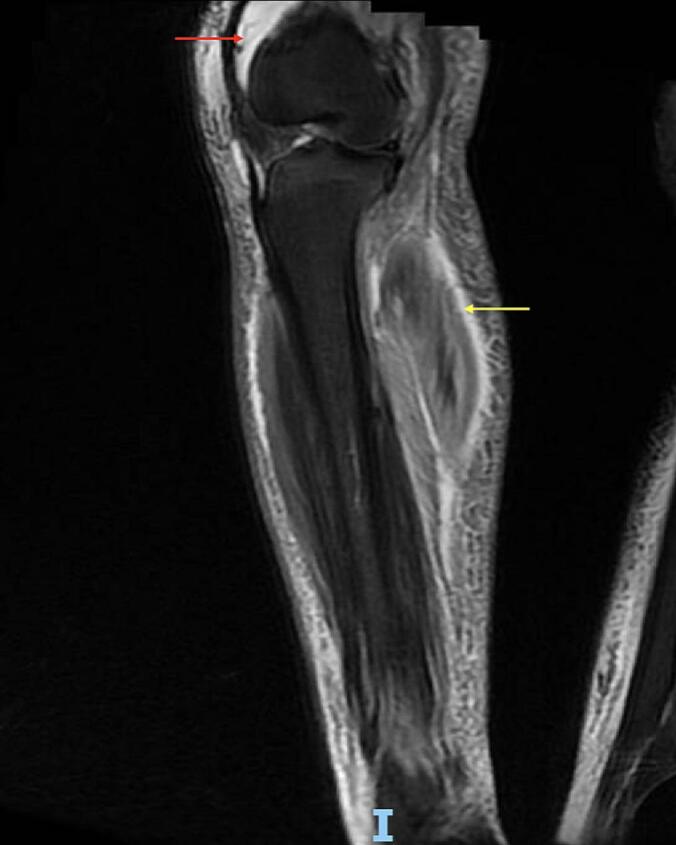
Sagittal T2-weighted MRI of Patient 1's right knee. The red arrow indicates a moderate suprapatellar joint effusion. The yellow arrow highlights a hyperintense signal within the posterior compartment musculature, overlaid by subcutaneous oedema and dermal thickening, consistent with soft tissue infection involving the fascia and muscle. (For interpretation of the references to colour in this figure legend, the reader is referred to the web version of this article.)

Despite initial improvement, the patient's posterior leg developed worsening swelling, erythema, and desquamation. MRI confirmed myositis and cellulitis, raising suspicion for NF (Fig. 1).

**Fig. 2:**
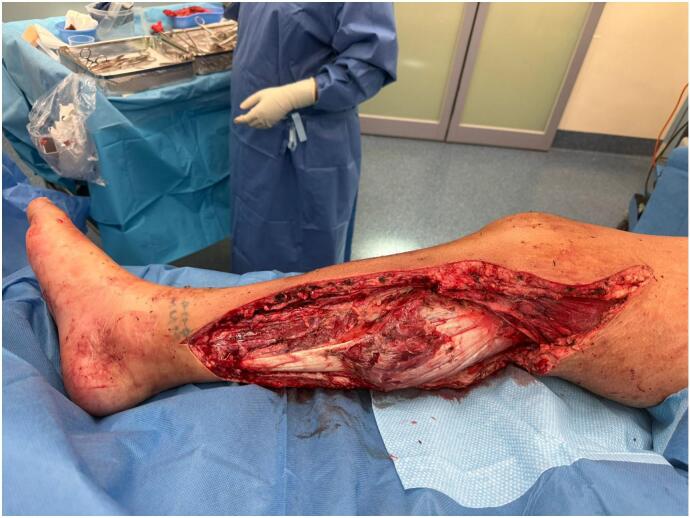
Intraoperative photograph of Patient 1 showing exposed musculature of the posterior leg compartment following excision of necrotic fascia and drainage of purulent material.

On postoperative day six, extensive debridement of necrotic fascia in the posterior leg was performed (Fig. 2), revealing purulent fluid tracking to the popliteal fossa. Two additional debridements were subsequently required. The resulting soft tissue defect, including exposed Achilles tendon, was managed with a vacuum-assisted closure (VAC) device and eventual application of a Biodegradable Temporising Matrix (BTM®) on day sixteen.

**Fig. 3:**
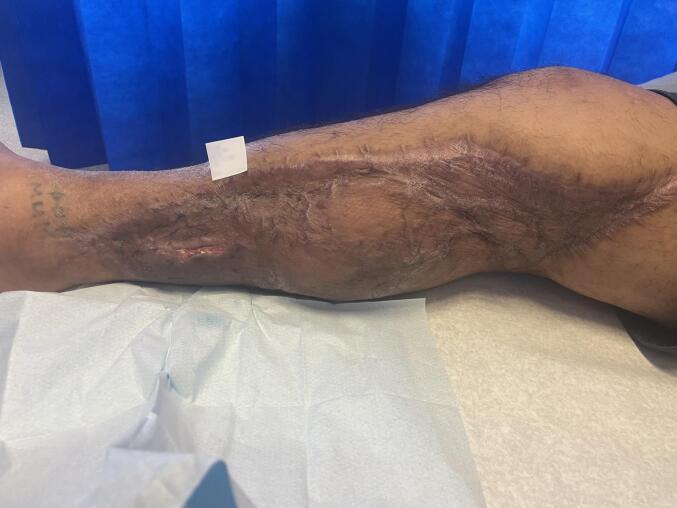
Clinical photograph of Patient 1's right lower limb approximately two months following split-thickness skin grafting over a biodegradable temporising matrix (BTM®). The graft demonstrates near-complete take, with a small area of healthy granulation tissue visible at the inferior aspect.

The patient improved clinically and was later transferred for rehabilitation. Approximately two months later, he underwent BTM delamination and split-thickness skin grafting to his leg. At follow-up two months post-grafting, the graft had taken well (Fig. 3), and the patient was independently weight-bearing.

## 3 Case 2

A 78-year-old male with poorly controlled type 2 diabetes mellitus and a history of left lentiform nucleus stroke presented to the emergency department with five days of right shoulder pain. His plain radiographs were also unremarkable. Initially diagnosed with cuff arthropathy and discharged, he returned four days later with delirium, lethargy, and worsening right upper limb swelling.

Examination revealed anterior arm cellulitis, with marked tenderness along the biceps sheath and an irritable shoulder. Laboratory investigations showed hyperglycaemia, and markedly elevated inflammatory markers. SA was diagnosed, and empirical intravenous piperacillin-tazobactam and clindamycin were initiated.

**Fig. 4:**
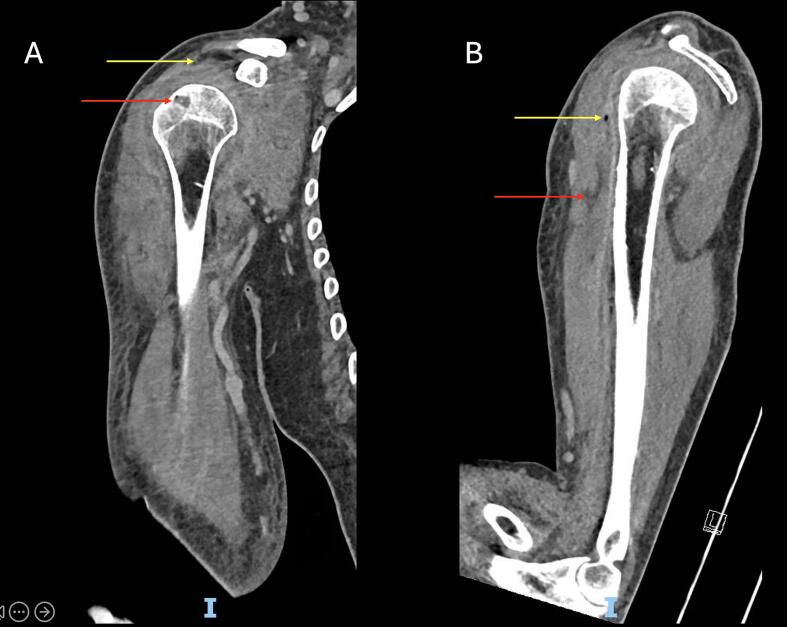
(A) Coronal CT image of Patient 2's right shoulder showing a gas-containing subacromial bursal effusion (yellow arrow) communicating with the glenohumeral joint via a full-thickness supraspinatus tear, consistent with septic arthritis. Note bone erosion and a small focus of intraosseous gas at the greater tuberosity (red arrow), consistent with osteomyelitis. (B) Sagittal CT image demonstrating a fluid collection (red arrow) with a gas locule (yellow arrow) tracking along the anterior compartment musculature of the arm, consistent with necrotising fasciitis. (For interpretation of the references to colour in this figure legend, the reader is referred to the web version of this article.)

CT imaging demonstrated gas within the glenohumeral joint, biceps tendon sheath, and subscapularis muscle, as well as osteomyelitis of the proximal humerus and concurrent NF of the anterior upper arm (Fig. 4). The patient received an urgent open right shoulder washout with exploration of the surrounding soft tissues.

**Fig. 5:**
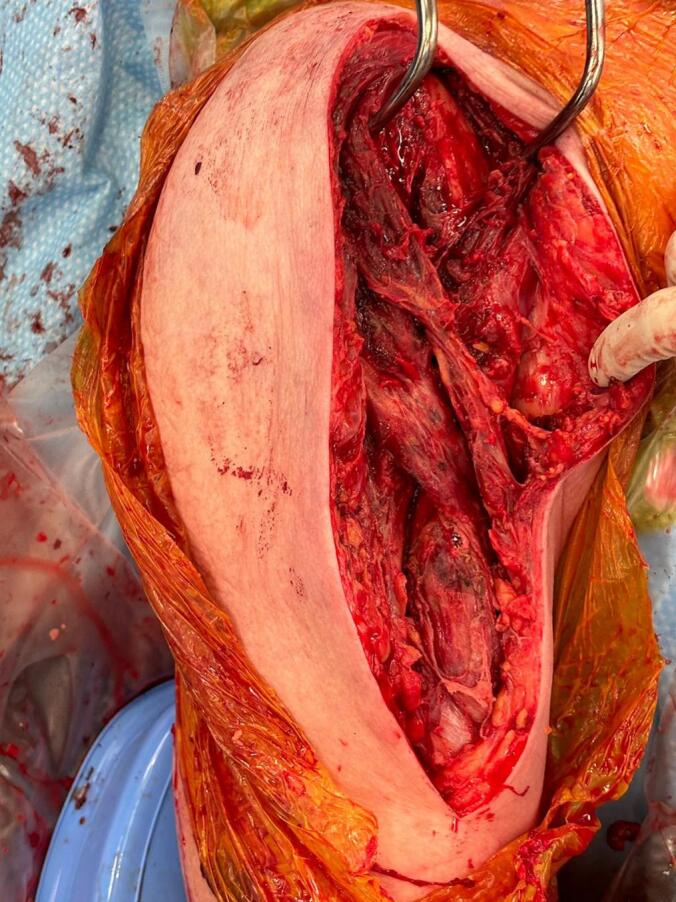
Intraoperative photograph of Patient 2 showing exposed musculature of the anterior arm following debridement of necrotic tissue and drainage of pus.

Pus was found in the subacromial, glenohumeral, sub-coracoid, and subdeltoid spaces, as well as within the biceps sheath. The proximal humerus required drilling due to osteomyelitis, and necrotic muscle in the anterior compartment was debrided (Fig. 5). Postoperatively, the patient required ICU admission due to septic shock. Antibiotics were later rationalised to benzylpenicillin following culture confirmation of Group B Streptococcus (GBS).

Ongoing soft tissue infection necessitated three further debridements. A VAC dressing was applied between procedures, and delayed primary wound closure was performed on day seven. The patient improved and was eventually transferred to the ward for continued antibiotic therapy, wound care, glycaemic control, and rehabilitation.

At follow-up two months after the final debridement and wound closure, the wound had healed, and shoulder function had returned to near baseline.

## 4 Discussion

Septic arthritis results from bacterial invasion of the synovial joint, typically via hematogenous spread, direct inoculation, or contiguous extension from a neighbouring infection [5].

Local soft tissue dissemination of septic arthritis, though rare, is a catastrophic complication that, through its progression to NF, increases mortality and prolongs hospitalisation [6,7]. The synovial membrane, despite being highly vascular, lacks a basement membrane and is vulnerable to bacterial invasion and further spread [8,9].

Patients particularly at risk of this sequela tend to possess factors that impair the immune response, disrupt joint integrity, or compromise synovial vascularity. These include advanced age, pre-existing joint pathology, prosthetic joints, and immunosuppressive conditions [10]. Diabetes mellitus with poor glycaemic control, a key risk factor in both cases, exacerbates susceptibility by impairing immune function and causing microvascular dysfunction [11].

As demonstrated by Case 2, delayed diagnosis – often due to the clinical overlap between septic arthritis and inflammatory or degenerative arthropathies – may precipitate SA dissemination, significantly worsening outcomes. Serum inflammatory markers should be routinely assessed in any patient presenting with significant monoarticular pain or acute loss of range of motion even in the absence of fever [12]. Extra-synovial extension of septic arthritis should be suspected when erythema and tenderness extend well beyond the soft tissue overlying the affected joint.

Importantly, NF may also precede and precipitate SA. The timeline of symptoms can offer clues to the pathogenesis. In patients where severe joint pain is the initial feature—as in the presented cases—SA is presumed to be the primary infection. However, in many instances, the sequence of events cannot be definitively established.

Arthrocentesis remains essential for definitive diagnosis of SA. Synovial fluid should be analysed for leukocyte count, Gram stain, and culture. A leukocyte count exceeding 50,000 cells/mm^3^ with a predominance of polymorphonuclear cells is highly suggestive of infection [ 10]. While Gram stain has limited sensitivity, its specificity is excellent and can provide early confirmation, enabling more targeted empirical antibiotic therapy. A positive culture remains the gold standard for diagnosis and antimicrobial stewardship [5,10].

Once septic arthritis is deemed the most likely diagnosis or confirmed, imaging can help assess the extent of disease prior to operative intervention. MRI is the gold standard for detecting both septic arthritis and soft tissue involvement, as plain radiographs are often unremarkable in early stages [13,14]. However, its use may be limited by access and availability in acute care settings. CT can assist in identifying joint or soft tissue changes but is less sensitive than MRI. Ultrasound can assess effusions and superficial soft tissue but is limited for deeper structures [13,14]. The decision to pursue pre-operative imaging – potentially delaying definitive surgical management – should be guided by clinical risk factors, timing of presentation, and resource availability.

Ideally, empirical intravenous antibiotics should be initiated after obtaining a synovial aspirate and performing a Gram stain to guide agent selection and minimise the risk of false-negative cultures [12,13]. However, treatment should not be delayed while awaiting Gram stain results, particularly in patients exhibiting signs of systemic sepsis. A combination of vancomycin with ceftazidime or an aminoglycoside offers broad coverage against most pathogens implicated in septic arthritis [12,15]. Antibiotic selection should also be guided by local resistance patterns, individual patient risk factors (e.g. MRSA, Pseudomonas, or gonococcal infection), and input from infectious diseases specialists. In cases with radiological evidence or strong clinical suspicion of soft tissue involvement, broader coverage targeting NF is warranted—typically a carbapenem or piperacillin-tazobactam plus vancomycin and clindamycin [16]. Therapy can then be narrowed once a causative organism is identified.

Arthroscopic or open debridement should be performed as early as possible to reduce bacterial load [13]. The choice of surgical approach should be guided by disease severity. The Gächter classification is commonly used, though not clinically validated [17]. Experts recommend arthroscopic debridement for Gächter stages I to III, with open debridement considered for stages III and IV [13]. In cases where extra-synovial extension is suspected or confirmed radiologically, an open approach is more appropriate. During the initial procedure, exploration of the peri-synovial region for evidence of dissemination is advisable. Serial debridements are often necessary in cases complicated by NF [ 18]. In cases with extensive tissue necrosis, amputation of the extremity may be the only viable option to achieve source control. Extensive local tissue necrosis and repeated surgical intervention typically result in large soft tissue defects requiring reconstruction. VAC dressings can be used temporarily until definitive coverage is achieved, which may involve skin grafts, locoregional flaps, or, in severe cases, free flaps [3]. Additionally, the destructive nature of this condition often necessitates prolonged rehabilitation, and many patients experience lasting functional impairment.

## 5 Conclusion

Septic arthritis with local soft tissue dissemination is a rare but devastating complication that increases the risk of limb loss and mortality. Early recognition, particularly in patients with risk factors such as diabetes or immunosuppression, is crucial. Prompt initiation of empirical antibiotics, appropriate use of imaging, and timely surgical intervention are essential to optimising outcomes.

## Author contribution

1. Ojochonu D Anthony
  Design of methodology, acquisition, analysis and interpretation of data, writing draft
2. Jevan Cevik
  Conceptualisation and design of study, analysis and interpretation of data, supervision, writing and critical revision of draft
3. Nelson Low
  Conceptualisation and design of study, analysis and interpretation of data, supervision, writing and critical revision of draft

## Informed consent

Written informed consent was obtained from the patient (or NOK) for publication of this case report and accompanying images. A copy of the written consent is available for review by the Editor-in-Chief of this journal on request.

## Ethical approval

At our institution, Monash Health – Dandenong Hospital, ethics approval is not required as the study is a descriptive report of 2 cases with no experimental intervention outside of standard care, patient confidentiality is protected and informed consent was obtained.

## Guarantor

Ojochonu Anthony, Jevan Cevik, Nelson Low.

## Research registration number

N/A.

## Funding

This research did not receive any specific grant from funding agencies in the public, commercial, or not-for-profit sectors.

## Conflict of interest statement

None.

## References

[1] Singh J.A., Yu S. (2018). Septic arthritis in emergency departments in the US: a national study of health care utilization and time trends. Arthritis Care Res..

[2] Windsor C., Hua C., De Roux Q., Harrois A., Anguel N., Montravers P. (2022). Healthcare trajectory of critically ill patients with necrotizing soft tissue infections: a multicenter retrospective cohort study using the clinical data warehouse of Greater Paris University Hospitals. Ann. Intensive Care.

[3] Somasundaram J., Wallace D.L., Cartotto R., Rogers A.D. (2021). Flap coverage for necrotising soft tissue infections: a systematic review. Burns.

[4] Kerwan A., Al-Jabir A., Mathew G., Sohrabi C., Rashid R., Franchi T., Nicola M., Agha M., Agha R.A. (2025). Revised Surgical CAse REport (SCARE) guideline: An update for the age of Artificial Intelligence. Prem. J. Sci..

[5] He M., Arthur Vithran D.T., Pan L., Zeng H., Yang G., Lu B. (2023). An update on recent progress of the epidemiology, etiology, diagnosis, and treatment of acute septic arthritis: a review. Front. Cell. Infect. Microbiol..

[6] Ferrand J., El Samad Y., Brunschweiler B., Grados F., Dehamchia-Rehailia N., Sejourne A. (2016). Morbimortality in adult patients with septic arthritis: a three-year hospital-based study. BMC Infect. Dis..

[7] Al-Qurayshi Z., Nichols R.L., Killackey M.T., Kandil E. (2020). Mortality risk in necrotizing fasciitis: national prevalence, trend, and burden. Surg. Infect..

[8] Haywood L., Walsh D.A. (2001). Vasculature of the normal and arthritic synovial joint. Histol. Histopathol..

[9] Mader J.T., Shirtliff M., Calhoun J.H. (1999). The host and the skeletal infection: classification and pathogenesis of acute bacterial bone and joint sepsis. Baillieres Best Pract. Res. Clin. Rheumatol..

[10] Margaretten M.E., Kohlwes J., Moore D., Bent S. (2007). Does this adult patient have septic arthritis?. JAMA.

[11] Berbudi A., Rahmadika N., Tjahjadi A.I., Ruslami R. (2020). Type 2 Diabetes and its Impact on the Immune System. Curr. Diabetes Rev..

[12] Earwood J.S., Walker T.R., Sue G.J.C. (2021). Septic arthritis: diagnosis and treatment. Am. Fam. Physician.

[13] Ravn C., Neyt J., Benito N., Abreu M.A., Achermann Y., Bozhkova S. (2023). Guideline for management of septic arthritis in native joints (SANJO). J Bone Jt Infect..

[14] Expert Panel on Musculoskeletal I, Pierce J.L., Perry M.T., Wessell D.E., Lenchik L., Ahlawat S. (2022). ACR appropriateness criteria(r) suspected osteomyelitis, septic arthritis, or soft tissue infection (excluding spine and diabetic foot): 2022 update. J. Am. Coll. Radiol..

[15] Benito N., Martinez-Pastor J.C., Lora-Tamayo J., Ariza J., Baeza J., Belzunegui-Otano J. (2024). Executive summary: guidelines for the diagnosis and treatment of septic arthritis in adults and children, developed by the GEIO (SEIMC), SEIP and SECOT. Enferm. Infecc. Microbiol. Clin. (Engl. Ed.).

[16] Sartelli M., Guirao X., Hardcastle T.C., Kluger Y., Boermeester M.A., Rasa K. (2018). 2018 WSES/SIS-E consensus conference: recommendations for the management of skin and soft-tissue infections. World J. Emerg. Surg..

[17] Gächter A. (1985). Der gelenkinfekt. Inform. Arzt..

[18] Anaya D.A., Dellinger E.P. (2007). Necrotizing soft-tissue infection: diagnosis and management. Clin. Infect. Dis..

